# Ophthalmic Solution of Smart Supramolecular Peptides to Capture Semaphorin 4D against Diabetic Retinopathy

**DOI:** 10.1002/advs.202203351

**Published:** 2022-11-27

**Authors:** Ya‐Nan Li, Hong‐Wen Liang, Chun‐Lin Zhang, Yan‐Mei Qiu, David Wang, Hai‐Ling Wang, An‐Qi Chen, Can‐Dong Hong, Lei Wang, Hao Wang, Bo Hu

**Affiliations:** ^1^ Department of Neurology Union Hospital Tongji Medical College Huazhong University of Science and Technology Wuhan 430022 China; ^2^ CAS Center for Excellence in Nanoscience CAS Key Laboratory for Biomedical Effects of Nanomaterials and Nanosafety National Center for Nanoscience and Technology (NCNST) Center of Materials Science and Optoelectronics Engineering University of Chinese Academy of Sciences Beijing 100190 China; ^3^ Neurovascular Division Department of Neurology Barrow Neurological Institute Saint Joseph's Hospital and Medical Center Phoenix AZ 85013 USA

**Keywords:** diabetic retinopathy, eye drop, semaphorin 4D, smart supramolecular peptide

## Abstract

Diabetic retinopathy (DR) is the leading cause of vision loss in working age population. Intravitreal injection of anti‐VEGF antibody is widely used in clinical practice. However, about 27% of patients show poor response to anti‐VEGF therapy and about 50% of these patients continue to have macular thickening. Frequent intravitreal injections of antibody may increase the chance of endophthalmitis and cause visual loss or even blindness once happened. Therefore, there is a greatly urgent need for novel noninvasive target to treat DR clinically. Here, the formulation of a smart supramolecular peptide (SSP) eye drop for DR treatment that is effective via specifically identifying and capturing soluble semaphorin 4D (sSema4D), a strongly pro‐angiogenesis and exudates factor, is reported. The SSP nanostructures encapsulate sSema4D so that all biological effects mediated by three receptors of sSema4D are inhibited, thereby significantly alleviating pathological retinal angiogenesis and exudates in DR. Moreover, it is found that combination of SSPs eye drop and anti‐VEGF injection shows better therapeutic effect over anti‐VEGF treatment alone. Overall, SSP eye drop provide an alternative and effective method for noninvasive treatment for DR.

## Graphical abstract

Diabetic retinopathy is the leading cause of vision loss in working age population. Here, the formulation of a smart supramolecular peptide (SSP) eye drop that can significantly decrease pathological retinal neovascularization and vascular leakage is reported. More importantly, it is found that combination of SSP eye drop and anti‐VEGF injection shows ≈1.5 times better therapeutic effect over anti‐VEGF treatment alone.

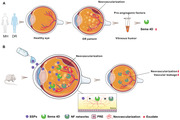

## 1 Introduction

Diabetic retinopathy (DR), one of the main and progressive complications of diabetes, is the leading cause of loss of vision in 20 to 79 years old population.^[^ ^1^ ^]^ Aggregate data showed that 103.12 million adults were estimated to be diagnosed with DR worldwide in 2020. About 629 million people are expected to have diabetes by 2045 globally.^[^ ^2^ ^]^ Most patients are diagnosed with DR 10 to 15 years after the onset of their diabetes. Finding an effective treatment of DR has become a challenge worldwide.^[^ ^3^ ^]^

Two most important pathogenesis of DR are neovascularization and exudates.^[^ ^4^ ^]^ Vascular endothelial growth factor (VEGF) elevated in DR, is a pro‐angiogenesis factor that promotes vascular angiogenesis and leakage.^[^ ^5^ ^]^ Therefore, intravitreal injection of anti‐VEGF antibody is widely use in clinical practice.^[^ ^5^ ^]^ However, about 27% of patients show poor response to anti‐VEGF therapy and 50% of these patients continue to have macular edema.^[^ ^6^ ^]^ Studies have confirmed that other factors (except for VEGF) may also play a role in accelerating pathological retinal angiogenesis and exudates and making anti‐VEGF ineffective.^[^ ^7^, ^8^ ^]^

Mountains of researches demonstrated that soluble semaphorin 4D (sSema4D) strongly promoted angiogenesis and exudates in tumors.^[^ ^9^, ^10^ ^]^ In this study, we first found sSema4D overexpression in the vitreous humor of 19 DR patients. We found Sema4D knockout significantly reduced pathological retinal vascular neovascularization in the model of oxygen‐induced retinopathy (OIR) and reduced vascular leakage in the streptozotocin (STZ)‐induced retinopathy model. Sema4D is therefore a promising target for DR therapy. Previously, we have found that intravitreal injection of anti‐sema4D reduced pathological retinal angiogenesis and exudates in DR.^[^ ^11^ ^]^ However, key problem remains to be resolved. Antibody can only be delivered by intravitreal injection due to its large molecular weight. Frequent intravitreal injections of antibody may increase the chance of endophthalmitis, cataracts and vitreous hemorrhage, and cause visual loss or even blindness once happened.^[^ ^12^, ^13^, ^14^ ^]^ Furthermore, intravitreal treatment is a technically challenging invasive operation and not easily performed in nontertiary medical centers.

**Figure 1:**
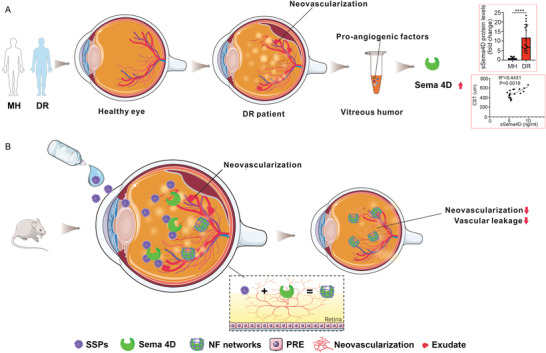
Elevated sSema4D level in the aqueous fluid of patients with DR and the schematic illustration of SSPs targeting sSema4D against diabetic retinopathy. A) The vitreous humors were acquired from patients with DR and macular hole (MH). Sema4D protein levels were significantly increased in the vitreous humor of patients with DR comparing to that in patients with MH. The correlation curves between the levels of sSema4D in aqueous fluid with changes of CST in response to anti‐VEGF therapy are shown. B) SSPs can noninvasively and effectively transfer into vitreous humor in the form of eye drop. By recognizing and encapsulating sSema4D, SSPs could inhibit Sema4D/PlexinB1 signal pathway which regulated pathological retinal neovascularization and vascular leakage in vitreous humor. DR, diabetic retinopathy; OIR, oxygen‐induced retinopathy; STZ, streptozotocin; SSP, smart supramolecular peptide.

Peptides with structural and biological functions have been widely used for disease therapeutics.^[^ ^15^, ^16^, ^17^ ^]^ Here, we report the formulation of a smart supramolecular peptide (SSP) eye drop for DR treatment that is effective via specifically identifying and capturing sSema 4D (**Figure** **1**). The sSema4D targeting SSP eye drops, SSP‐1 and SSP‐2, are based on one‐bead‐one‐compound (OBOC) combinatorial peptide library and “in vivo self‐assembly” concept. SSP‐1 and SSP‐2 self‐assembled into nanostructures first and in situ transform into nanofibers upon binding to sSema4D. Harnessing the advantage of self‐assembly into fibrous networks via induction of ligand‐receptor interaction, the targeting proteins can be identified and captured as well. SSP‐1 and SSP‐2 recognized and encapsulated sSema4D, leading to the failure of the combination of sSema4D and its receptors (including PlexinB1, PlexinB2 and CD72), which improved blocking efficiency. Moreover, we found that combination of SSP‐1 or SSP‐2 eye drop and anti‐VEGF injection showed ≈1.5 times better therapeutic effect over anti‐VEGF treatment alone. The “assembly/aggregation induced retention (AIR)” effect in specific sites in vivo may achieve prolonged retention for noninvasive treatment for DR.

## 2 Results

Targeting sSema 4D, we first designed SSP that can specifically identify and capture sSema4D protein. The SSP was comprised of three functional domains: 1) The targeting peptide was first discovered from OBOC combinatorial peptide library; 2) FFVLK derived from *β*‐amyloid (A*β*) that as *β*‐sheet‐forming peptide; 3) the palmitoleic acid (C16) as a hydrophobic unit, enhanced the hydrophobicity of peptide molecules, in addition, C16 once cleaved from SSP shows high biocompatiblity. SSP can pre‐assemble into nanostructures, such as spherical nanoparticles (NPs) or short‐rod nanofibers (NFs), as shown in Figure 3B. The SSP nanostructures may bind to sSema4D and transform into fibrous networks in situ, trapping sSema4D. The SSP nanostructures then inhibited the combination of sSema4D and its receptors, alleviating its pathological retinal angiogenesis and exudates in DR. More importantly, SSP nanostructures can function as an eye drop, decreasing injection induced damage.

### 2.1 sSema4D Expression is Increased in the Vitreous Humor of DR Patients and Library Screening and Binding Affinity

**Figure 2:**
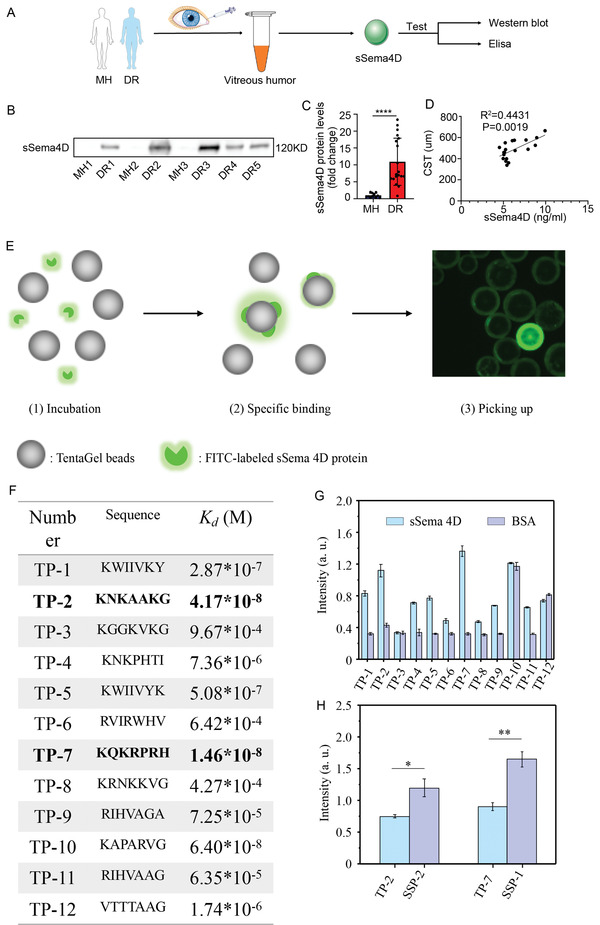
sSema4D expression is increased in the vitreous humor of DR patients and library screening and binding affinity. A) sSema4D protein levels in the vitreous humor from MH patients or DR patients were evaluated by Western blot or ELISA. B,C) Representative immunoblots and quantification analysis of sSema4 protein levels in the vitreous humor from DR patients and MH patients (*n* = 12 for MH group, and n = 19 for DR group). D) sSema4 protein levels are positively correlated with CST in DR patients (*n* = 19). E) Schematic illustration of screening from OBOC library. F) SPRi of the binding affinity of TPs to sSema 4D. G) ELISA of binding affinity of TPs to sSema 4D and BSA. H) ELISA of binding affinity of TPs and SSPs to sSema 4D. TPs, transformable peptides; SPRi, surface plasmon resonance imaging; BSA, bovine serum albumin, OBOC, one‐bead one‐compound; SSP, smart supramolecular peptide.

To explore the potential role of sSema4D in the pathogenesis of human DR, we first examined sSema4D protein levels in the vitreous humor of patients with DR, as shown in **Figure** **2**A and Table S1 (Supporting Information). Samples of control were collected from vitreous humor of nondiabetic patients receiving surgery for idiopathic macular hole. Western blot revealed that sSema4D protein levels were significantly increased in the vitreous humor of patients with DR comparing to that in patients with macular hole (MH) in Figure 2B,C. Moreover, the protein levels of sSema4D were positively correlated with the central subfield thickness (CST) before receiving anti‐VEGF therapy in Figure 2D.

sSema4D has a ligand‐binding extracellular domain to bind to its specific receptors on cell membrane and promotes tumor progression through tumor angiogenesis and regulation of tumor‐associated macrophages. Screening the TP that can bind sSema4D and block the combination of sSema4D‐PlexinB1, sSema4D‐PlexinB2 and sSema4D‐CD72 is key to improve patients’ visual acuity and reduce associated complications. The OBOC combinatorial library was constructed through combinational synthesis using seven different natural amino acids as building blocks.^[^ ^15^ ^]^ Since one TentaGel S resin bead contains one peptide, the OBOC combinatorial library has a theoretical diversity of 18^7 coding peptides.

To carry out the library screening, we first labeled the sSema4D with fluorescein isothiocyanate isomer (FITC), as shown in Figure 2E. Next, ≈10^6 beads library was incubated with FITC‐labeled sSema4D for 6 hours. The beads were thoroughly washed with DMF, water and methanol. Finally, under the fluorescence microscope, the beads with weak or no binding to sSema4D or FITC‐labeled sSema4D through noncovalent bonding were identified in Figure 2F. We selected twelve strongest putative fluorescent beads by physical separation. Subsequently, the corresponding peptides were cleaved off with BrCN from beads and analyzed by automatic Edman sequencing. The structures and sequence of transformable peptides (TPs) were determined and summarized (Figure S2, Supporting Information). TP‐1 to TP‐12 were resynthesized into solid phase and purified by high performance liquid chromatography (HPLC). The binding affinity of TPs toward sSema 4D was measured by enzyme‐linked immunosorbent assay (ELISA) and surface plasmon resonance imaging (SPRi). Among them, TP‐2, TP‐7, and TP‐10 showed excellent binding affinity to sSema4D with a *K*_d_ value of 41.7 × 10^−9^, 14.6× 10^−9^, and 64.0 × 10^−9^ m in Figure 2G. However, TP‐2 and TP‐7 had more specific binding affinity to sSema4D protein compared to TP‐10 on ELISA and both bind with bovine serum albumin (BSA) protein in Figure 2H. TP‐2 and TP‐7 were selected as the candidate TP binding sSema 4D to construct SSP. Subsequent SSP monomers (SSP‐1, sequence: C16‐FFVLK‐KQKRPRH; SSP‐2 C16‐FFVLK‐KNKAAKG) were synthesized and characterized by HPLC and Matrix‐assisted laser desorption/ionization time‐of‐flight mass spectrometry (MALDI‐TOF‐MS). These SSPs, SSP‐1 and SSP‐2, showed higher binding affinity compared with TPs on ELISA that measured binding affinity with sSema4D in Figure 2H.

### 2.2 Self‐Assembly and NFs Transformation by Recognized sSema4D in Solution

**Figure 3:**
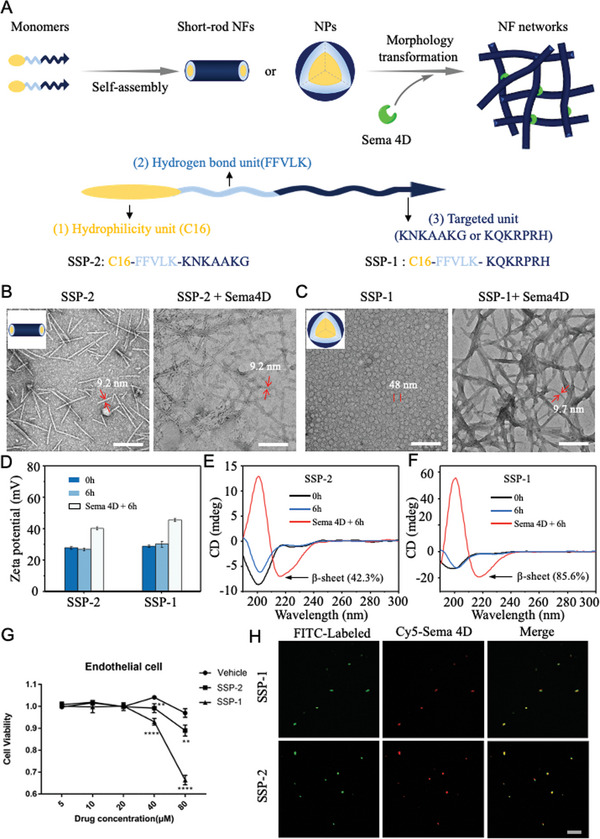
Assembly and transformation of SSP. A) Schematic illustration of self‐assembly and sSema 4D‐triggered morphology transformation. B, C) TEM images of initial short‐rod NFs (SSP‐1) and NPs (SSP‐2) transformed into NF networks after incubation with Sema 4D protein for 6 h (sSema 4D:SSP = 1:1000, mass ratio). Scale bars, 200 nm. Zeta potential (D), circular dichroism spectra (E, F) of SSPs with and without Sema 4D. G) Cell viability of SSP. H) CLSM showing specific recognition and capture of SSP (greeen) to sSema 4D (red). Scale bar, 20 µm. All experiments were repeated three times. NPs, nanoparticles; NFs, nanofibers; SSP, smart supramolecular peptide.

Artificial tears containing SSPs were developed and dropped into eyes. SSPs accumulated in the vitreous cavity. To examine the behavior of SSPs monomer in artificial tear, it was fast injected into water. Under the aqueous condition, the SSPs self‐assembled into spherical NPs or nanorods, a function of its to C16 and FFVLK domains with a hydrophobic unit. The morphology of SSP‐1 was NPs with the average size of 44.8 ± 2.9 nm. The transmission electron microscope (TEM) exhibited that the average length of SSP‐2 nanorods was 190 ± 23.9 nm (**Figure** **3**B). Besides, the zeta potential of SSP‐1 and SSP‐2 was positive charged with the value of 45.7 and 40.2 mV, respectively (Figure 3C). The positive charged SSPs penetrate blood‐eye barrier well.

To investigate the interaction of sSema4D with SSPs, SSP‐1 and SSP‐2, were incubated with the sSema4D in solution at room temperature. The mass ratio of sSema4D protein/SSPs was 1/1000, and it facilitated the formation of the NFs network. We found that SSPs morphology was unchanged for 72 h before interacting with sSema4D protein (Figure S1, Supporting Information). After incubation with sSema4D for 6 h, SSP‐2 showed a long‐size of nanofibrillar structures. However, SSP‐1 exhibited a dense NFs network structures as shown in Figure 3B. These results demonstrated that the binding of SSP‐1 or SSP‐2 to sSema4D and the morphology of SSP‐1 or SSP‐2 can transform NPs or nanorods to NFs or network.

In addition, KLVFF sequence, a sheet structure with strong hydrogen‐bonding was introduced into SSPs, we could distinctly observe the secondary structure of SSPs by circular dichroism (CD) spectroscopy. As shown in Figure 3D, CD signal for SSP‐1 or SSP‐2 changed before and after incubation with sSema4D. After incubation with sSema4D protein for 4 h, both SSP‐1 and SSP‐2 showed a positive signal at 215 nm and a negative signal at 200 nm, indicative of a typical *β*‐sheet structure. The percentage of *β*‐sheet after incubation with sSema4D in SSP‐1 and SSP‐2 were 85.6% and 42.3%, respectively, indicative of a highly organized packing of NFs in SSP‐1. The zeta potential of SSP‐1 and SSP‐2 was almost unchanged during 0 or 6 h without sSema4D, however, it remarkably increased to 45.7 and 40.2 for SSP‐1 and SSP‐2 after incubation with sSema4D, respectively (Figure 3C). This zeta potential indicated that more positive charged residues had a morphological transformation. These results further revealed that the recognized interaction of SSP‐1 and SSP‐2 to sSema4D destroyed the dynamic balance of the original state of SSP‐1 and SSP‐2, and formed into thermodynamically stable NFs. The high percentage of *β*‐sheet structure in NFs stabilized the formation of nanostructure networks, due to its strong hydrogen‐bonding KLVFF sequence. Moreover, we carried out the cytotoxicity of SSP‐1 and SSP‐2 by using a standard CCK‐8 (Figure 3D). The viability of mouse brain microvascular endothelial cells (MBMEC) is close to 90% after the incubation with SSP‐1 and SSP‐2 for 24 h, even at concentrations up to 80 × 10^−6^ m, suggesting that SSP‐1 and SSP‐2 nearly had no clear toxicity (Figure 3E). Finally, we investigated the recognition of sSema4D and SSP‐1 and SSP‐2 by confocal laser scanning microscope (CLSM).

FITC‐labeled SSP‐1 or SSP‐2 and Cy5‐labeled sSema4D proteins were synthesized. Cy5‐labeled sSema4D were incubated with FITC‐labeled SSP‐1 and SSP‐2 nanostructure solutions for 6 h, respectively. As shown in Figure 3F, FITC‐labeled SSP‐1 and SSP‐2 showed green fluorescence signals, and Cy5‐labeled sSema 4D proteins showed red fluorescence signals. Merge images suggested that SSP‐1 and SSP‐2 could completely cover the sSema4D, indicating excellent recognition and trapping capability of SSP‐1 and SSP‐2 of sSema4D protein.

### 2.3 sSema4D Expression is Increased in the STZ Model and Sema4D Knockout Reduces Pathological Retinal Neovascularization and Vascular Leakage

**Figure 4:**
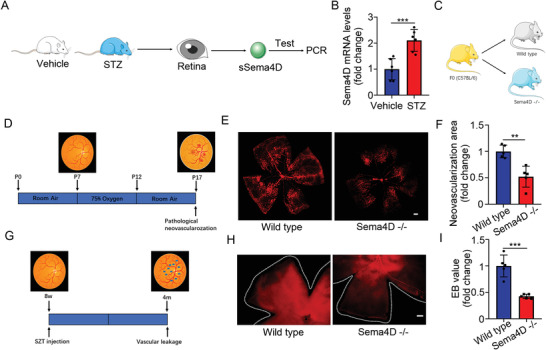
sSema4D expression is increased in the STZ model and Sema4D knockout reduces pathological retinal neovascularization and vascular leakage. A,B) sSema4D mRNA levels in the retina from the STZ model were evaluated by PCR (*n* = 6). C) Schematic illustration for generation of Sema4D knockout mice. D) Schematic illustration depicting OIR model establishment. E, F) Sema4D knockout reduces pathological retinal neovascularization in the OIR model (*n* = 5). G) Schematic illustration depicting STZ model establishment. H, I) Sema4D knockout reduces retinal vascular leakage in the STZ model (*n* = 5). All data are represented as mean ± SD. *p < 0.05, **p < 0.01, ***p < 0.005, ****p <0.0001. DR, diabetic retinopathy; MH, macular hole; OIR, oxygen‐induced retinopathy; STZ, streptozotocin; CST, central subfield thickness.

We established two different models for DR. STZ‐induced mouse DR model (STZ model) developed only retinal vascular leakage without neovascularization. OIR model developed pathological neovascularization. In the STZ mouse model, which mainly mimics retinal vascular leakage of DR, we found that the mRNA levels of sSema4D was increased (**Figure** **4**A,B). To explore the role of Sema4D in DR, we generated Sema4D knockout mice (Figure 4C). Isolectin B4 staining showed that Sema4D knockout significantly reduced pathological retinal vascular neovascularization in the OIR model (Figure 4D–F). Evans blue assays showed that Sema4D knockout reduced retinal vascular leakage in the STZ model (Figure 4G–I).

### 2.4 Intraocular Injection of SSP‐1 and SSP‐2 Alleviate Abnormal Retinal Neovascularization and Vascular Leakage In Vivo

**Figure 5:**
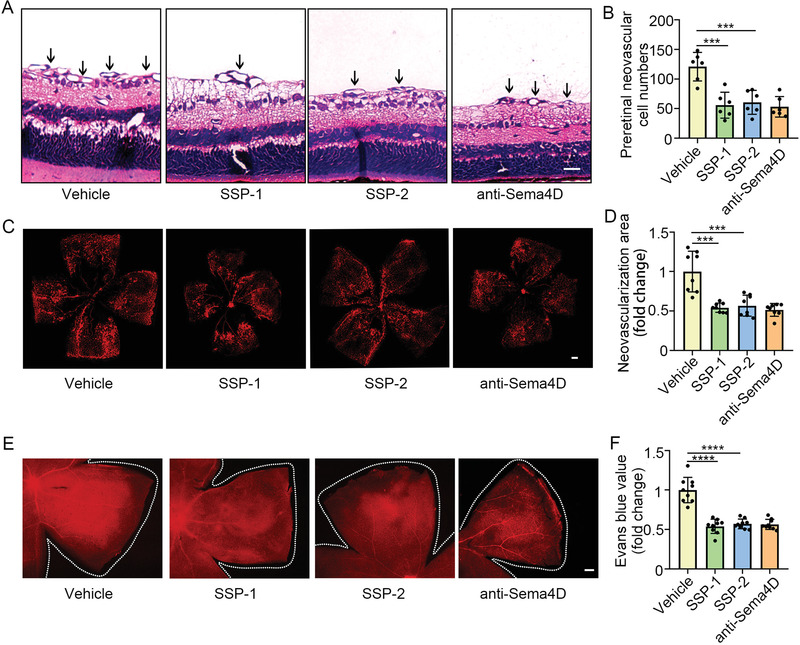
Intraocular injection of SSP‐1 and SSP‐2 alleviate abnormal retinal neovascularization and vascular leakage in vivo. A,B) The OIR model was intravitreally injected with control solvent or SSP‐1 or SSP‐2 at P12, and the retinas were harvested at P17. Intraocular injection of SSP‐1 or SSP‐2 significantly reduced the preretinal neovascular cell numbers in the OIR model compared with littermate controls. Scale bars indicate 20 µm (*n* = 6). C, D) Intraocular injection of SSP‐1 or SSP‐2 significantly reduced abnormal neovascularization through isolectin B4 staining compared with littermate controls. Scale bars indicate 200 µm (*n* = 8). E,F) In the STZ model, mice were intravitreally injected with control solvent or SSP‐1 or SSP‐2 at the third month since diabetes induction, and the retinas were harvested at the fourth month. The Evans blue assay showed SSP‐1 or SSP‐2 significantly reduced retinal vascular leakage. Scale bars indicate 200 µm (*n* = 9). All data are represented as mean ± SD. *p < 0.05, **p < 0.01, ***p < 0.005, ****p < 0.0001. OIR, oxygen‐induced retinopathy; STZ, streptozotocin.

To study the role of SSP‐1 and SSP‐2 in alleviating pathological retinal vascular neovascularization and vascular leakage, we performed intravitreal injection of SSP‐1 and SSP‐2 in both OIR models and STZ models. SSP‐1 and SSP‐2 significantly reduced the numbers the preretinal neovascular cell compared with littermate controls in OIR model (**Figure** **5**A,B). Moreover, isolectin B4 staining demonstrated that SSP‐1 and SSP‐2 decreased pathological retinal vascular neovascularization in the OIR model (Figure 5C,D). In general, the effects of SSP‐1 and SSP‐2 on reducing pathological retinal vascular neovascularization were equal to that of Sema4D antibody. To study the role of SSP‐1 and SSP‐2 in diabetic retinal vascular leakage, we performed Evans blue assays. As shown in Figure 5E,F, SSP‐1 and SSP‐2 significantly decreased vascular leakage in the STZ model. The effects of SSP‐1 and SSP‐2 on alleviating diabetic retinal vascular leakage and acellular capillary formation were similar to anti‐Sema4D antibody.

### 2.5 SSP‐1 and SSP‐2 Regulate Endothelial Cell Function and Pericyte Function In Vitro

**Figure 6:**
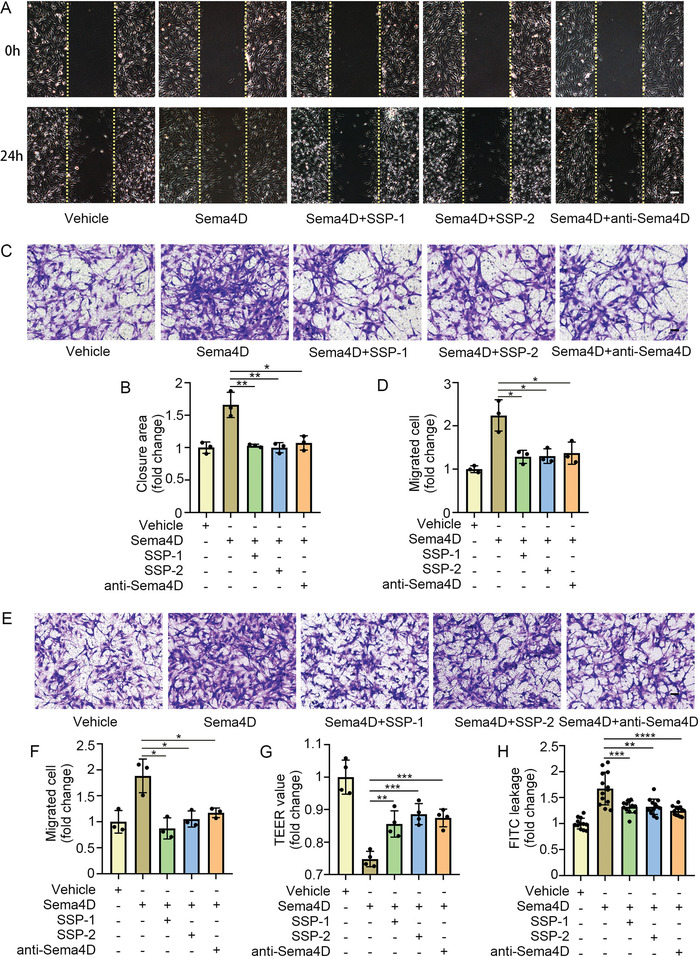
SSP‐1 and SSP‐2 regulate endothelial cell function and pericyte function in vitro. A,B) Wound healing assays showed that SSP‐1 and SSP‐2 inhibited Sema4D‐induced endothelial cell migration. Scale bars indicate 100 µm (*n* = 3). C,D) Transwell migration assays showed that SSP‐1 and SSP‐2 inhibited Sema4D‐induced endothelial cell migration. Scale bars indicate 100 µm (*n* = 3). E,F) Transwell migration assays showed that SSP‐1 and SSP‐2 reduced Sema4D‐induced pericyte migration. Scale bars indicate 100 µm (*n* = 3). G,H) Trans‐endothelial electrical resistance (TEER) value and fluorescein isothiocyanate isomer (FITC) permeability were measured to evaluate the effect of SSP‐1 and SSP‐2 on the barrier function of the co‐culture model of primary endothelial cells with pericytes (*n* = 4 for TEER value measurement and *n* = 13 for FITC permeability measurement). All data are represented as mean ± SD. *p < 0.05, **p < 0.01, ***p < 0.005, ****p <0.0001. FITC, fluorescein isothiocyanate isomer; TEER, trans‐endothelial electrical resistance.

Endothelial cell and pericyte exert an essential role in DR. We investigated the effects of SSP‐1 and SSP‐2 on endothelial cell function and pericyte function in vitro. Wound‐healing assays and transwell assays indicated that SSP‐1 and SSP‐2 inhibited recombinant Sema4D protein‐induced endothelial cell migration, the effects of which were equal to that of anti‐Sema4D antibody (**Figure** **6**A–D). Furthermore, SSP‐1 and SSP‐2 reduced recombinant Sema4D protein‐induced pericyte migration (Figure 6E,F). To investigate the function of SSP‐1 and SSP‐2 in vascular permeability in vitro, TEER and permeability to dextran were performed in a co‐culture model of primary endothelial cells with pericytes. We found that recombinant Sema4D protein damaged vascular permeability of the co‐culture model of primary endothelial cells with pericytes, which was partly reversed by SSP‐1 and SSP‐2 (Figure 6G,H). The effects of SSP‐1 and SSP‐2 on alleviating damaged vascular permeability were similar to anti‐Sema4D antibody.

### 2.6 Intraocular Delivery of SSP‐1 and SSP‐2 In Vivo and Vitro

**Figure 7:**
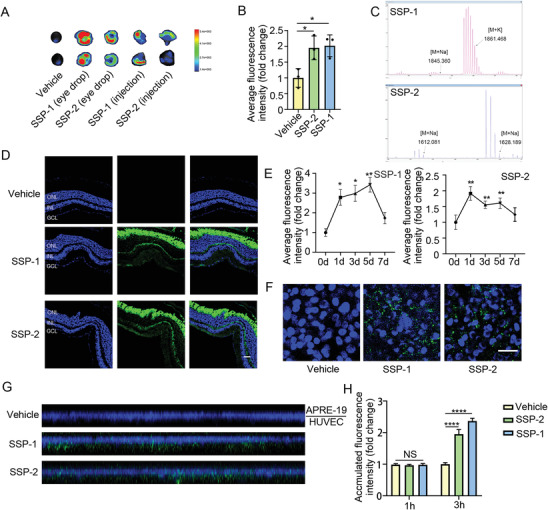
Intraocular delivery of SSP‐1 and SSP‐2 in vivo and vitro. A) Bioluminescence images of the eyes receiving FITC‐labeled SSP‐1 or SSP‐2 eye drops. B) Average fluorescence intensity of the vitreous humor from the mice receiving FITC‐labeled SSP‐1 or SSP‐2 eye drops (*n* = 3, each sample was mixed from 5 mice's vitreous humor). C) Representative chromatograms of SSP‐1 and SSP‐2 in vitreous humor. D) Intraocular distribution of FITC‐labeled SSP‐1 and SSP‐2 in the retinas from the mice receiving FITC‐labeled SSP‐1 and SSP‐2 eye drops. Scale bars indicate 100 µm. E) Average fluorescence intensity of the vitreous humor from the mice receiving FITC‐labeled SSP‐1 or SSP‐2 eye drops at different time points (*n* = 3, each sample was mixed from 5 mice's vitreous humor). F) Representative fluorescence images of FITC‐labeled SSP‐1 or SSP‐2 in the bottom HUVEC layer. Scale bars indicate 20 µm. G) 3D fluorescence images of FITC‐labeled SSP‐1 or SSP‐2 in the in vitro BRB. The upper side was the ARPE‐19 cell layer and the bottom side was the HUVEC layer. H) Cumulative amount of FITC‐labeled SSP‐1 or SSP‐2 permeating across the in vitro BRB model after incubation for 1 h and 3 h (*n* = 16). All data are represented as mean ± SD. *p < 0.05, **p < 0.01, ***p < 0.005, ****p < 0.0001. FITC, fluorescein isothiocyanate isomer; HUVEC, human umbilical vein endothelial cells; ARPE‐19, adult retinal pigment epithelial cell line‐19.

To study the intraocular distribution of the FITC‐labeled SSP‐1 and SSP‐2 after topical instillation, fluorescence observation and liquid chromatography‐mass spectrometry (LC/MS) were performed on the entire eyes of mice, the frozen sections of the eyes, and their vitreous humor (**Figure** **7**A–D). After 12 h FITC‐labeled SSP‐1 or SSP‐2 were instilled, the treated eye emitted a distinct fluorescence signal, mainly in the retina and vitreous humor (Figure 7A,B,D). The topical instillation of SSP eye drop was instilled once with 10 µL before fluorescence observation. However, intraocular injection was administrated once with the maximum volume of 2 µL to avoid overdose induced high intraocular pressure due to the limited volume of the vitreous cavity. The different dose for the two routes of administration may explain why that topical instillation of SSP displayed much more accumulation of SSP in eye than that intraocular injection. Next, we studied the intraocular pharmacokinetics of SSP‐1 and SSP‐2 after topical instillation. As shown in Figure 7E, fluorescence observation was performed on the mice vitreous humor at different time points. SSP‐1 and SSP‐2 showed their presence up to 5 days in the mice vitreous humor. These results indicated that SSP‐1 and SSP‐2 showed their long‐time retention (up to 5 days) in the mice vitreous humor. Moreover, we established an in vitro blood‐retina barrier (BRB) model to investigate the transferring efficiency of SSP‐1 and SSP‐2 across the posterior ocular absorption barriers. As shown in Figure 7F, fluorescence images of FITC‐labeled nanoparticle distribution in the bottom human umbilical vein endothelial cells (HUVEC) layer indicated that green fluorescence was observed in both SSP‐1 and SSP‐2 treated layers. The 3D images of the BRB side view clearly showed the distribution of SSP‐1 and SSP‐2 in the in vitro BRB model in Figure 7G. Obvious green fluorescence was observed in both SSP‐1 and SSP‐2 treated BRB in the upper adult retinal pigment epithelial cell line‐19 (ARPE‐19) layer and bottom HUVEC layer, which indicated that SSP‐1 and SSP‐2 effectively transferred across the BRB in Figure 7H.

### 2.7 SSP‐1 and SSP‐2 Eye Drops Reduce In Vivo Pathological Retinal Neovascularization and Vascular Leakage

**Figure 8:**
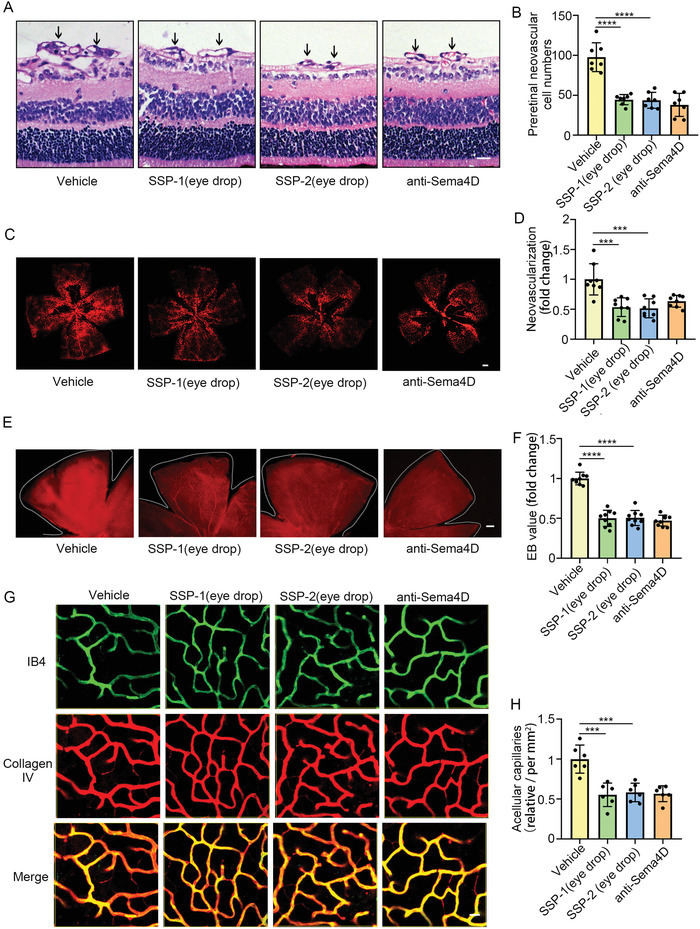
SSP‐1 and SSP‐2 eye drops reduce pathological retinal neovascularization in and vascular leakage in vivo. A,B) The OIR model was treated with control solvent or SSP‐1 or SSP‐2 eye drops after P12, which was twice a day until P17. SSP‐1 and SSP‐2 eye drop significantly reduced the preretinal neovascular cell numbers in the OIR model. Scale bars indicate 20 µm (*n* = 7). C,D) SSP‐1 and SSP‐2 eye drop significantly reduced abnormal neovascularization through isolectin B4 staining. Scale bars indicate 200 µm (*n* = 8). E,F) In the STZ model, mice were treated with control solvent or SSP‐1 or SSP‐2 eye drops at the third month since diabetes induction, which was twice a day and lasted for 30 d. The Evans blue assay showed SSP‐1 or SSP‐2 significantly reduced retinal vascular leakage. Scale bars indicate 20 µm (*n* = 9). G, H) Immunofluorescence staining of PDGFRb with collagen IV in the STZ model. Scale bars indicate 10 µm (*n* = 6). All data are represented as mean ± SD. *p < 0.05, **p < 0.01, ***p < 0.005, ****p < 0.0001. OIR, oxygen‐induced retinopathy; STZ, streptozotocin.

Next, we investigated the therapeutic effects of SSP‐1 and SSP‐2 eye drops on DR in the OIR model and the STZ model. In the OIR model, administration of 5 µL per eye of artificial tear fluid or of SSP‐1 and SSP‐2 eye drops was performed twice daily from P12 to P17. After the administration of SSP‐1 and SSP‐2 eye drops during the period up to 6 d, SSP‐1 and SSP‐2 significantly decreased the numbers of preretinal neovascular cells compared with littermate controls (**Figure** **8**A,B). Moreover, isolectin B4 staining demonstrated that SSP‐1 and SSP‐2 decreased pathological retinal vascular neovascularization (Figure 8C,D). In the STZ model, administration of 10 µL per eye of artificial tear fluid or of SSP‐1 and SSP‐2 eye drops was performed twice daily for 30 d after the third month since diabetes induction. Evans blue assays indicated that SSP‐1 and SSP‐2 eye drops significantly decreased vascular leakage in the STZ model (Figure 8E,F). Furthermore, SSP‐1 and SSP‐2 ameliorated diabetes‐induced retinal acellular capillary formation (Figure 8G,H). In general, the effects of SSP‐1 and SSP‐2 eye drops on reducing pathological retinal vascular neovascularization and vascular leakage were equal to that of intraocular injection of anti‐Sema4D antibody.

### 2.8 Biosafety of SSP‐1 and SSP‐2 in the Retina

**Figure 9:**
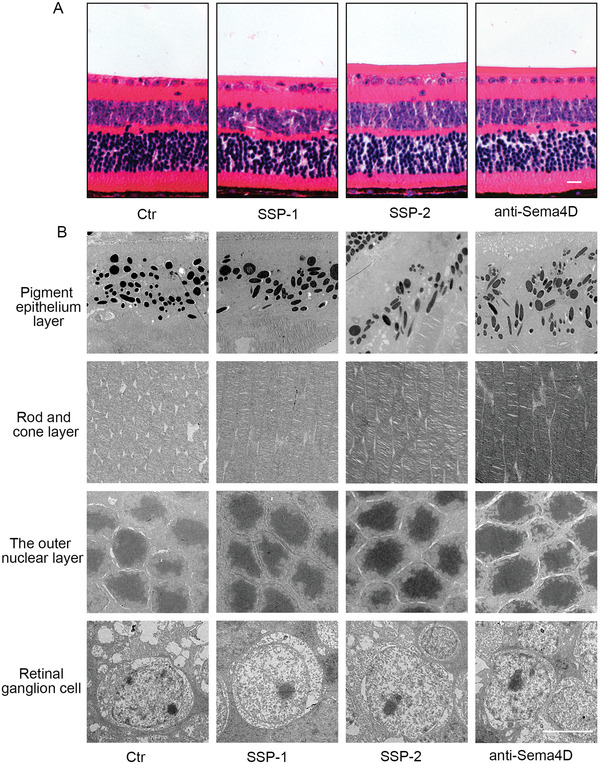
Safety of SSP‐1 and SSP‐2. A) HE staining of whole retinas receiving intraocular injection of SSP‐1 and SSP‐2. Scale bars indicate 20 µm. B) Representative electron microscopy images of different retinal cells of mice receiving intraocular injection of SSP‐1 and SSP‐2. Scale bars indicate 20 µm. Scale bars indicate 5 µm.

After 30 d of SSP‐1 and SSP‐2 intraocular injection, the biosafety of SSP‐1 and SSP‐2 was systematically studied by H&E staining and electron microscopy. As shown in **Figure** **9**A, tissue sections stained with HE showed no significant pathological changes in the retina. The electron microscopy images revealed no significant abnormalities in retinal pigment epithelium, rod and cone layer, the outer nuclear layer and retinal ganglion cells in Figure 9B.

### 2.9 Anti‐VEGF and SSP‐1 and SSP‐2 Eye Drops Have Synergistic Effect on Alleviating Pathological Retinal Neovascularization and Vascular Leakage

**Figure 10:**
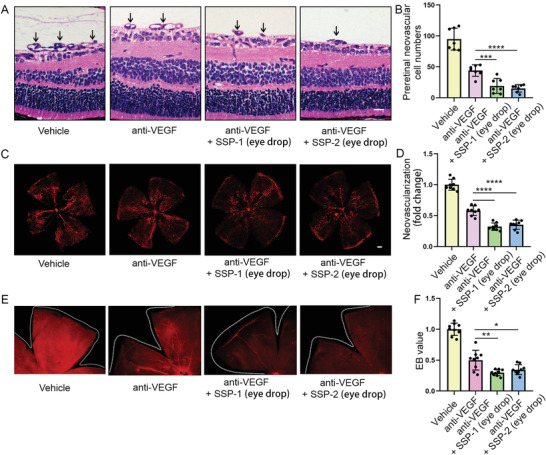
Anti‐VEGF and SSP‐1 and SSP‐2 eye drops have synergistic effect in alleviating pathological retinal neovascularization and vascular leakage. A–D) The OIR model was intravitreally injected with anti‐VEGF antibody (2 µg) at P12. At the same time, the mice were treated with control solvent or SSP‐1 or SSP‐2 eye drops after P12, which was twice a day until P17. The combination of SSP‐1 or SSP‐2 eye drops with intraocular injection anti‐VEGF antibody (2 µg) have synergistic effect in reducing pathological retinal neovascularization in the OIR model (*n* = 7 for B, *n* = 8 for D). E,F) In the STZ model, mice were intravitreally injected with anti‐VEGF antibody (2 µg) at third month, and treated with control solvent or SSP‐1 or SSP‐2 eye drops, which was twice a day and lasted for 30 d. The combination of SSP‐1 or SSP‐2 eye drops with intraocular injection anti‐VEGF antibody (2 µg) have synergistic effect in reducing retinal vascular leakage (*n* = 9). All data are represented as mean ± SD. *p < 0.05, **p < 0.01, ***p < 0.005, ****p < 0.0001. OIR, oxygen‐induced retinopathy; STZ, streptozotocin.

Anti‐VEGF therapy is a standard treatment for DR. However, some patients are refractory to these treatments, about 40% of patients show poor response to anti‐VEGF therapy and continue to have macular thickening. Previously, we found that SSP‐1 and SSP‐2 eye drops reduced pathological retinal neovascularization and vascular leakage in the OIR model and STZ model. Next, we explored whether a combination of anti‐VEGF injection and SSP‐1 and SSP‐2 eye drops would provide an enhanced therapeutic effect. Isolectin B4 staining showed that SSP‐1 eye drops plus anti‐VEGF injection reduced pathological retinal neovascularization by 25.72% on average as compared with anti‐VEGF injection alone, and the effect of SSP‐2 eye drops plus anti‐VEGF injection increases 22.68% on average as compared with anti‐VEGF injection (**Figure** **10**A–D). Moreover, Evans blue assay indicated that SSP‐1 eye drops plus anti‐VEGF injection reduced retinal vascular leakage by 20.21% on average than anti‐VEGF injection alone, and the effect of SSP‐2 eye drops plus anti‐VEGF injection increases 15.49% on average than anti‐VEGF injection based on Figure 10E,F.

## 3 Discussion

In this study, we collected the vitreous humor of 19 patients with DR and found that the expression of sSema 4D was significantly increased. Sema4D knockout significantly reduced pathological retinal vascular neovascularization and leakage in the OIR and STZ model. More importantly, we designed SSPs, which utilized in situ ligand‐receptor–induced self‐assembly that could recognize and trap sSema4D in vivo. When compared with anti‐Sema 4D antibody, SSPs at 20 µM displayed similar therapeutic effects. Our in vitro data clearly demonstrated that SSP‐1 and SSP‐2 could capture the sSema4D and downregulate expression of sSema 4D. SSP‐1 and SSP‐2 can be administered as eye‐drops and showed high transferring efficiency and therapeutic benefit, indicating a high clinical applicability. In addition, the combination of SSP‐1 and SSP‐2 and anti‐VEGF showed a better therapeutic effect over monotherapy. It is useful for patients with less or no response to anti‐VEGF therapy since about 40% patients are resistant to anti‐VEGF.^[^ ^18^ ^]^

Hetian Lei confirmed that in addition to VEGF, there may be other pro‐angiogenic molecules in the aqueous humor circulation.^[^ ^7^, ^8^ ^]^ Therefore down‐regulating VEGF levels alone cannot achieve a complete therapeutic effect which may be the reason why anti‐VEGF treatment only works for some patients. In our study of 19 patients with diabetic retinopathy, expression of sSema 4D correlated with the leakage of the fundus. We established two pathological models, OIR and STZ, to simulate the pathological neovascularization and leakage of the fundus in diabetic retinopathy. We found that the expression of retinal sSema 4D was significantly increased in both OIR model and STZ model. Sema4D knockout significantly reduced pathological retinal vascular neovascularization and leakage in the OIR and STZ model.

Sema4D, also known as CD 100, belongs to transmembrane semaphorin class IV, which can be proteolytic cleaved to soluble form (sSema4D) and shed from the cell surface. sSema4D, a 120 kDa biologically active fragment, has a ligand‐binding extracellular domain to bind to its specific receptors on cell membrane.^[^ ^19^ ^]^ Studies have confirmed that sSema4D is an important molecule in colorectal and breast cancer and ischemic stroke to promote angiogenesis and leakage.^[^ ^20^, ^21^ ^]^ Our previous study also confirmed for the first time that anti‐Sema4D intravitreal administration can effectively inhibit fundus pathological angiogenesis and leakage in OIR and STZ models.^[^ ^11^ ^]^ Based on the solid study, sSema4D may be an important target to inhibit pathological angiogenesis and vascular leakage in DR. The safety and tolerability of intra‐venous injection of VX15/2503 (an antibody anti‐sema4D) in patients with advanced solid tumors have been confirmed.^[^ ^20^ ^]^ This therapeutic target of inhibiting angiogenesis and leakage maybe has precious clinical translation value.

However, administration of anti‐sema4D remains a challenge for clinical application. First, the large molecular size (size ≈150–160 kDa) of monoclonal Sema4D antibody can only be administered invasively by retrobulbar injection. Frequent intravitreal administrations have certain risks in inducing endophthalmitis, cataracts and vitreous hemorrhage, which will lead to potential vision loss even blindness.^[^ ^12^, ^13^ ^]^ Second, intravitreal administration demands high‐technology invasive operation.^[^ ^13^ ^]^ Furthermore, and the economic expenditures on antibody agents is huge.

First, we tried to find a sSema4D targeting peptide based on OBOC combinatorial peptide library, which provides a targeted screening method to discover peptides of interest (POI) with high specificity and affinity for targeted protein.^[^ ^17^ ^]^ We previously developed a rapidly fluorescent‐activation screening method to explore self‐assembly short peptides based on OBOC combinatorial technology.^[^ ^15^ ^]^ This simple screening strategy rapidly provides the possibility to obtain POI, the development of POI for various biomedical and material applications has been largely accelerated.^[^ ^22^ ^]^ The 10 sequences for targeting sSema4D were discovered, and both G and H with top 2 binding affinity were screened out in the second round.

In order to increase the possibility of penetration into eyes, facilitate therapeutic binding affinity and deliver therapeutic benefit, the in vivo self‐assembling concept was further introduced to design the peptide. Recently, “in vivo self‐assembly” concept as a strategy was proposed as an in situ construction of nanostructures in vivo in a smart way with “AIR” in vivo effect in specific sites.^[^ ^23^, ^24^ ^]^ Therefore, we combined the screened targeting sequence with beta‐sheet forming sequence and alkyl chain to form the SSP, SSP‐1 and SSP‐2 through peptide bonds. As expected, the SSP‐1 and SSP‐2 self‐assembled into nanostructures first and in situ transform into nanofibers upon binding to sSema4D. The fibrous networks in turn could trap the sSema4D with long‐term and multi‐valance effect. As a result, the SSP, SSP‐1 and SSP‐2 showed similar therapeutic efficacy with much lower dose compared with antibody.

As anticipated, the SSP targeting sSema4D, SSP‐1 and SSP‐2 can penetrate the blood‐retina barrier when delivered in the eye drop and showed in vivo inhibition of sSema4D. The penetration was achieved due to its small molecular weight (SSP‐2,1.59k Da; SSP‐1,1.82k Da) and positive zeta potential. In detail, SSP‐1 and SSP‐2 in the form of eye drops are incredibly capable of penetrating the ocular surface into the vitreous humor. Fluorescence and LC/MS detected SSP‐1 and SSP‐2 mainly in the retina and vitreous humor at 12 h after SSP‐1 and SSP‐2 instillation. SSP‐1 and SSP‐2 was still present up to 5 d in the mice vitreous humor.

There are three receptors known for sSema4D: high‐affinity receptor Plexin‐B1 (PlxnB1), intermediate affinity Plexin‐B2 (PlxnB2), and low‐affinity receptor CD72.^[^ ^25^ ^]^ Our previous study found that sSema4D promoted angiogenesis and leakage through binding to the PlexinB1 receptor.^[^ ^11^ ^]^ sSema4D also bond to PlexinB2 or CD72 to mediate the proinflammatory responses, which may promote angiogenesis and leakage indirectly.^[^ ^26^ ^]^ Therefore, completely blocking the binding of sSema4D to any receptor can achieve the best therapeutic effect. SSP‐1 and SSP‐2 recognize and encapsulate sSema4D so all biological effects mediated by three receptors of sSema4D were inhibited.

SSP‐1 and SSP‐2 nanostructures‐based eye drops show comparative or even better effect than injection of anti‐Sema4D to treat DR. The therapeutic effects of SSP‐1 and SSP‐2 eye drops on reducing pathological retinal vascular neovascularization and vascular leakage were equal to that of intraocular injection of anti‐Sema4D antibody in the OIR and STZ models. Mechanically, SSP‐1 and SSP‐2 significantly decreased sSema4D protein‐induced endothelial cell and pericyte migration, and protected the blood‐retina barrier function damaged by sSema4D protein. If combined with anti‐VEGF antibody in the treatment of neovascularization and leakage, it was even more effective. IB staining showed that SSP‐1eye drops plus anti‐VEGF injection reduced pathological retinal neovascularization by 25.72% on average as compared with anti‐VEGF injection alone, and the effect of SSP‐2 eye drops plus anti‐VEGF injection increases 22.68% on average as compared with anti‐VEGF injection. Moreover, Evans blue assay indicated that SSP‐1 eye drops plus anti‐VEGF injection reduced retinal vascular leakage by 20.21% on average than anti‐VEGF injection alone, and the effect of SSP‐ eye drops plus anti‐VEGF injection increases 15.49% on average than anti‐VEGF antibody injection.

## Conflict of Interest

The authors declare no conflict of interest.

## Supporting information

Supporting Information

## Data Availability

The data that support the findings of this study are available in the supplementary material of this article.
