# Supplementary material for: Ophthalmic Solution of Smart Supramolecular Peptides to Capture Semaphorin 4D against Diabetic Retinopathy
Source: Adv Sci (Weinh). 2022 Nov 27;10(3):2203351. doi: 10.1002/advs.202203351 (PMC9875641; doi:10.1002/advs.202203351)
Supplement: Supplementary file 1 — Supporting Information [file ADVS-10-2203351-s001.pdf]

ADVANCED SCIENCE
Open Access

Supporting Information

for *Adv. Sci.*, DOI 10.1002/advs.202203351

Ophthalmic Solution of Smart Supramolecular Peptides to Capture Semaphorin 4D against Diabetic Retinopathy

Ya-Nan Li, Hong-Wen Liang, Chun-Lin Zhang, Yan-Mei Qiu, David Wang, Hai-Ling Wang, An-Qi Chen, Can-Dong Hong, Lei Wang, Hao Wang* and Bo Hu**

Supporting Information

Ophthalmic Solution of Smart Supramolecular Peptides to Capture Semaphorin 4D Against Diabetic Retinopathy

Ya-Nan Li, Hong-Wen Liang, Chun-Lin Zhang, Yan-Mei Qiu, David Wang, Hai-Ling Wang, An-Qi Chen, Can-Dong Hong, Lei Wang, Hao Wang*, Bo Hu**

Affiliations:

Dr. B. Hu, Y.-N. Li, C.-L. Zhang, Y.-M. Qiu, H.-L. Wang, A.-Q. Chen, C.-D. Hong
Department of Neurology, Union Hospital, Tongji Medical College, Huazhong University of Science and Technology; Wuhan 430022, China.
Prof. H. Wang, L. Wang, H.-W. Liang
CAS Center for Excellence in Nanoscience, CAS Key Laboratory for Biomedical Effects of Nanomaterials and Nanosafety, National Center for Nanoscience and Technology (NCNST), Center of Materials Science and Optoelectronics Engineering, University of Chinese Academy of Sciences; Beijing 100190, China.
Prof. D. Wang
Neurovascular Division, Department of Neurology, Barrow Neurological Institute, Saint Joseph's Hospital and Medical Center; Phoenix, Arizona. Department of Neurology, Co-Director of Vascular Neurology and Petznick Stroke Center, Deputy Editor & General Manager, Stroke and Vascular Neurology.

*Corresponding author Email: hubo@hust.edu.cn, wanghao@nanoctr.cn and wanglei@nanoctr.cn

MATERIALS AND METHODS

Materials. TentaGel S Resin was purchased from Rapp Polymere (Germany, loading 0.31 mmol/g). 9-Fluorenylmethoxycarbonyl (Fmoc)-protected amino acids,

2-(1H-benzotriazole-1-yl)-1,1,3,3- tetramethyluronium hexafluoro-phosphate (HBTU), and Wang resin were obtained from GL Biochem (China). Trifluoroacetic acid (TFA), fluorescein isothiocyanate (FITC), N-methyl morpholine (NMM), piperidine, and N,N′-dimethylformamide (DMF) were all from Beijing Chemical Plant (China). Cyanogen bromide (CNBr) was from J&K Chemical (China). The OBOC pentapeptide libraries (19^5) were built using Fmoc strategy SPPS (solid-phase peptide synthesis) according to reported literature. TP-1 to TP-12, SSP-1 and SSP-2 were purchased by GL Biochem (Shanghai) Ltd.

Preparation of peptide-based nanomaterials. SSP-1 or SSP-2 monomers were dissolved in artificial tears at a concentration of 4.0 mM and sonicated for 30 min, followed by quick injection into water at a volume ratio of 1:200 for artificial tears and H_2O to obtain the nanomaterials solution (20 μM).

TEM for the morphology. The SSP-1 or SSP-2 solution with the concentration of 20 μM (10 μL) was placed dropwise onto a copper mesh for 5 min, then most of the liquid was removed through a filter paper. Uranyl acetate solution (10 μL) were employed to stain the samples for 5 min, followed by drying the spare liquid with the filter. Finally, the copper mesh was washed with 10 μL of deionized water, which was blotted after staining and drying at room temperature. All of the samples were observed by TEM (Tecnai G2 20 S-TWIN) at an accelerating voltage of 120 kV.

CD spectra. The CD spectra of SSP-1 or SSP-2 (30 μM) were collected at room temperature using a CD spectrometer (JASCO-1500, Tokyo, Japan) with a cell path length of 1 mm. The measurements were implemented between 190 and 230 nm with

a resolution of 1.0 nm and a scanning speed of 300 nm/min. For each measurement, three spectra were collected and averaged.

CLSM observation. Sema4D protein was purchased from Novoprotein Ltd. (Suzhou, China). Sema4D protein cultured with SSP-1 or SSP-2 nanomaterials (30 μM) for 4 h were imaged using a Zeiss LSM710 confocal laser scanning microscope (Ultra-VIEWVox, PerkinElmer). The parameters of confocal laser scanning microscope was a 40× objective lens.

Patient. Vitreous humor samples of DR patients were obtained immediately before intravitreal injection of anti-VEGF agents. Control vitreous humor samples were collected from nondiabetic patients receiving surgery for idiopathic macular hole. All of the human vitreous humor samples were collected based on informed consent. The vitreous samples were centrifuged at 4°C, and the supernatants were aliquoted into several sterile tubes and stored at -80°C. The procedure was approved by an ethics committee of Tongji Medical College, Huazhong University of Science and Technology, and strictly followed the Declaration of Helsinki guidelines.

Cell culture. The cerebral cortex of a 3-week-old mouse was minced and digested with 0.1% type II collagenase at 37°C for 45 min. The suspension was centrifuged at 1500g for 15 minutes and the pellet was resuspended in 33% Percoll medium. After the suspension was centrifuged at 1000 g at 4°C for 10 minutes, the microvessel fragments suspended in the middle layer of the culture medium were collected. Endothelial cells were cultured in Medium 131 (Invitrogen, Carlsbad, CA, USA), and

pericytes were cultured in Pericyte Medium (ScienCell, USA). bEnd.3 cells were purchased from ATCC and grown in DMEM with 10% fetal bovine serum.

Preparation method of nanoparticles. The nanomaterials FG-12 and FH-12 were added to a 1:1 mixture of propylene glycol and ethanol solution to form a 2 mM mother liquor. The mother solution was diluted in normal saline at 1:100 dilution to form a 20 μM nanoparticle solution. The artificial tears used in our experiments are Polyethylene Glycol Eye Drops (Alcon Laboratories, Inc), also known as Systane Ultra Lubricant Eye Drops. Its main ingredients are polyethylene glycol and propylene glycol. For cell experiments, the vehicle group is DMSO plus Medium 131 (primary endothelial cells), DMSO plus Pericyte Medium (primary pericytes) or DMSO plus DMEM (bEnd.3 cells). For animal experiments with SSPs intraocular injection, the vehicle group is DMSO plus physiological saline. For animal experiments with SSPs eye drop, the vehicle group is DMSO plus artificial tears.

CCK8 assay. Primary endothelial cells were seeded in the 96-well plate. After 24 h incubation with FH (5, 10, 20, 40, 80 nM) or FG (5, 10, 20, 40, 80 nM), 10 μL CCK8 solution was added. After 2 h incubation with CCK8 solution at 37℃ in a 5% CO2 atmosphere, the fluorescence intensity was evaluated by a fluorescence microplate reader (PerkinElmer, Waltham, MA, USA).

real-time PCR. Total RNAs were extracted from the mice retinas using Trizol reagent (Vazyme, Nanjing, China). RNA retrotranscription was performed with the HiScript II Q RT SuperMix (Vazyme, Nanjing, China), and cDNA was amplified

with the AceQ qPCR SYBR Green Master Mix (Vazyme, Nanjing, China). Primers are listed below:

Gene	Forward (5'-3')	Reverse (5'-3')
Sema4D	CCTGGTGGTAGTGTTGAGAAC	GCAAGGCCGAGTAGTTAAAGAT
β-actin	CCAGTTGGTAACAATGCCATGT	GGCTGTATTCCCCTCCATCG

Western blot. Protein samples from vitreous humor were subjected to 10% polyacrylamide gel electrophoresis. The gel-separated proteins were transferred onto the polyvinylidene difluoride (PVDF) membranes. After blocked with 5% skim milk, the membranes were incubated with antibodies against Sema4D (R&D system) or β-actin (Abclonal) overnight at 4°C. After washing, the membranes were incubated with horseradish peroxidase-conjugated secondary antibody for 2h at room temperature, and visualized by ECL solution (Abclonal) on a BioSpectrum Imaging System (UVP, Upland, CA, USA). The densitometry was quantified using ImageJ.

Would healing assay. The fused endothelial cells were starved in DMEM without FBS for 6 hours, and a sterile 200 μL pipette tip was used to create a cross linear scratch. After washing the floating cells with PBS, the cell culture medium of different stimulation groups was changed to DMEM containing mitomycin-C (10 μg/ml) and 0.5% FBS. At 0h and 12h, pictures of the wounds of endothelial cells were collected using a microscope. Use ImageJ to analyze the wound area.

Transwell assays. A 24-transwell dish (8-μm pore size, Corning, NY, USA) was used. After endothelial cells or pericytes were starved for 6h in DMEM containing 0.5% FBS, endothelial cells or pericytes were seeded to the upper chambers and

incubated for 8 h with serum-free DMEM with recombinant Sema4D protein (1600 ng/ml) or FH (20 nM) or FG (20 nM) in the lower chambers. After fixed with 4% PFA, the cells were stained with crystal violet solution for 10 min. The migrated cells were counted using a microscope with a digital camera.

Transendothelial electrical resistance (TEER) measurement and dextran permeability. The co-culture model of primary endothelial cells with pericytes was used for TEER measurement and dextran permeability assay. Endothelial cells were seeded on the lower chambers of transwell membranes and pericytes were seeded on the upper chambers (0.4-μm pore size, Corning, NY, USA). After the cells were co-cultured for 3 days, TEER measurements were performed after 24 h incubation with Sema4D protein (1600 ng/ml) or FH (20 nM) or FG (20 nM) via an Epithelial Volt/ohm Meter and STX2 electrodes (World 2 Precision Instruments, Sarasota, FL, USA).

FITC-labeled dextran (Sigma-Aldrich) was used for dextran permeability assay. After 24 h incubation with recombinant Sema4D protein (1600 ng/ml) or FH (20 nM) or FG (20 nM), FITC-dextran (300 μg/ml) was added into the upper chambers. 1 h after FITC-dextran was added, 50 μL liquid were taken from the lower chambers. The fluorescence intensity was measured by a fluorescence microplate reader (PerkinElmer, Waltham, MA, USA).

Elisa. Soluble Sema4D (sSema4D) levels in human vitreous humor samples or cell culture conditional medium were evaluated using ELISA kits (MyBioSource) according to the manufacturer’s instructions.

Transport of FG/FH across the in vitro BRB model. The in vitro BRB model was built as previously described before. ARPE-19 were seeded on the lower side of transwell membranes and HUVEC were seeded on the upper side (0.4-μm pore size, Corning, NY, USA). TEER measurements were performed via an Epithelial Volt/ohm Meter and STX2 electrodes (World 2 Precision Instruments, Sarasota, FL, USA). TEER value over 100 Ω·cm2 were selected for further experiments. After 24 h incubation with recombinant Sema4D protein (1600 ng/ml) or FH (20 nM) or FG (20 nM), FITC-dextran (300 μg/ml) was added into the upper chambers. 1 h after FITC-dextran was added, 50 μL liquid were taken from the lower chambers. The fluorescence intensity was measured by a fluorescence microplate reader (PerkinElmer, Waltham, MA, USA).

Mice. All animal experiment protocols were approved by medical Ethics Committee and Institutional Animal Care and Use Committee of Tongji Medical College, Huazhong University of Science and Technology (HUST), Wuhan, China. All mice were purchased and raised in the professional facilities of animal Feeding and Use Committee of Tongji Medical College of HUST, and were randomly assigned to each experimental group.

CRISPR/Cas9 system were used to generate Sema4D knockout mice as previously reported. The guide RNA (sgRNA) was designed online according to previous reported Sema4D-KO mouse (http://crispr.mit.edu). sgRNA and Cas9 mRNA was injected into the embryos to generate F0 mice, and adult F0 mice (8 weeks old) were mated to get offspring. The primers used for Genomic DNA identifying are as

follows: 5'-TCTGGGGCTCTAAGAGGTCCTT-3', 5'-AGCCACTGAGGTCACATACACC-3'.

STZ. Eight-week-old male C57BL/6 mice were starved for 4 hours before each injection of streptozotocin (STZ, Sigma, St. Louis, MO), and then injected with STZ (50 mg/kg) for 5 consecutive days. Mice in the vehicle control group were intraperitoneally injected with citrate buffer for 5 consecutive days. A blood glucose meter (ONETOUCH, Johnson & Johnson, USA) was used to evaluate the fasting blood glucose of the mice 7 days after the last injection of STZ. Mice with blood glucose levels greater than or equal to 16.7 mM were considered successful in STZ modeling. And monthly fasting blood glucose was monitored to exclude mice that failed to model.

OIR. OIR model was performed as previously described. The pups and their mothers were exposed to 75% oxygen at 7-12 days after birth, and then exposed to indoor air for another 5 days. At P17, the mice were anesthetized and sacrificed, and the retinas were harvested.

Isolectin B4. Isolectin B4 staining for retinal blood vessel visualization is as described above. After fixing the eyeball with 4% paraformaldehyde (overnight at 4 degrees), the intact retina was peeled off using microscissors and micro tweezers. After rupture of the membrane with 2% Triton X-100, the retina was placed in the configured isolectin B4 staining (1:100) overnight at 4 degrees. Acquire multiple overlapping (10-20% overlap) images using a fluorescence microscope and merge

them together in Adobe Photoshop to visualize the entire retina. Use ImageJ software to quantify the area of pathological neovascularization relative to the total retinal area.

Evans blue assay. Mice were intraperitoneally injected with Evans Blue dye (200 mg/kg) and circulated for 5 hours. The mice were perfused with PBS, and the eyes were fixed with 4% PFA at 4 degrees Celsius for 12 hours, and the retina was separated. Some retinas are tested for Evans Blue fluorescence using a fluorescence microscope. Adding formamide (70 μL/piece) to the rest of the retina and incubate overnight at 70°C to extract the formamide from the retina. Detect the absorbance of formamide at 620/740 nm (background) with a microplate reader. Calculate the content of Evans blue in the retina from the standard curve, and then standardize it based on the weight of the retina and the content of Evans blue in the blood.

Intraocular Distribution of FG/FH. Male mice (20-25 g) derived from Charles River (Beijing, China) were used in this experiment. 10 μL FITC-labeled FH or FG (dissolved in artificial tear fluid, 20 nM) were gently dripped in the conjunctival sac. The upper and lower eyelids were massaged to make homodisperse on the eye surface. After 12h FITC-labeled FH or FG were instilled, the mice were sacrificed and the eyeballs were wash in the PBS solution. luminescence signal was detected via the In-Vivo FX PRO system (Bruker).

Statistical analysis. Statistical analysis was conducted using Graphpad Prism 8.0 software. All data were expressed by mean ± SD. Unpaired two-tailed Student’s t-test (two groups) or one-way ANOVA (more than 2 groups) was used to analyze the statistical significances of the data. Person’s correlation analysis was conducted to

study the correlation between two variables. It was considered that $p < 0.05$ was significant, $p > 0.05$ was not significant. *$p < 0.05$, **$p < 0.01$, ***$p < 0.001$, ****$p < 0.0001$.

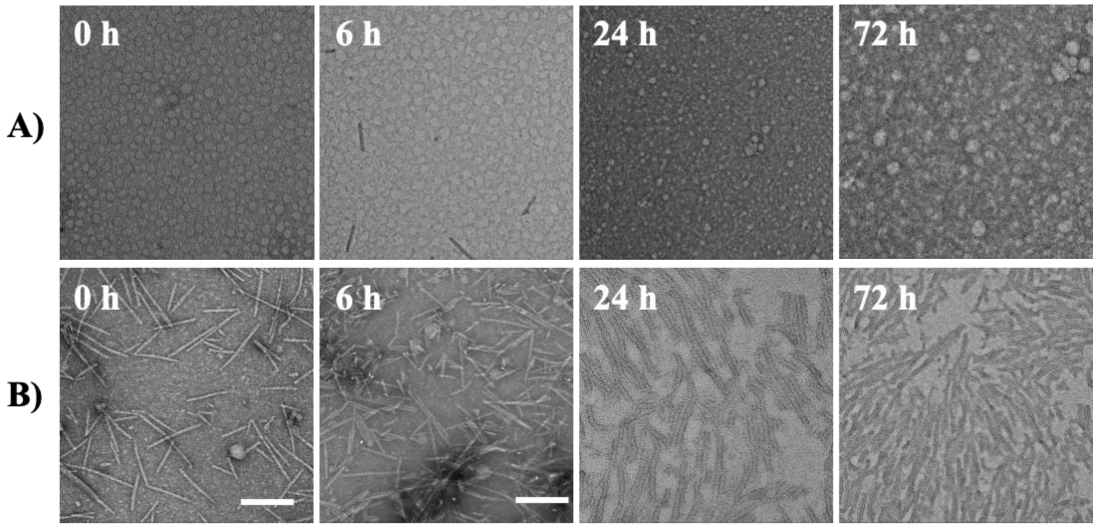

Figure S1. TEM images of freshly prepared SSP-1(A) and SSP-2 (B) after 6, 24, 72 h in PBS solution. The concentration of FG used in the experiment was 20μM. The scale bar is 100 nm. Experiments were repeated three times.

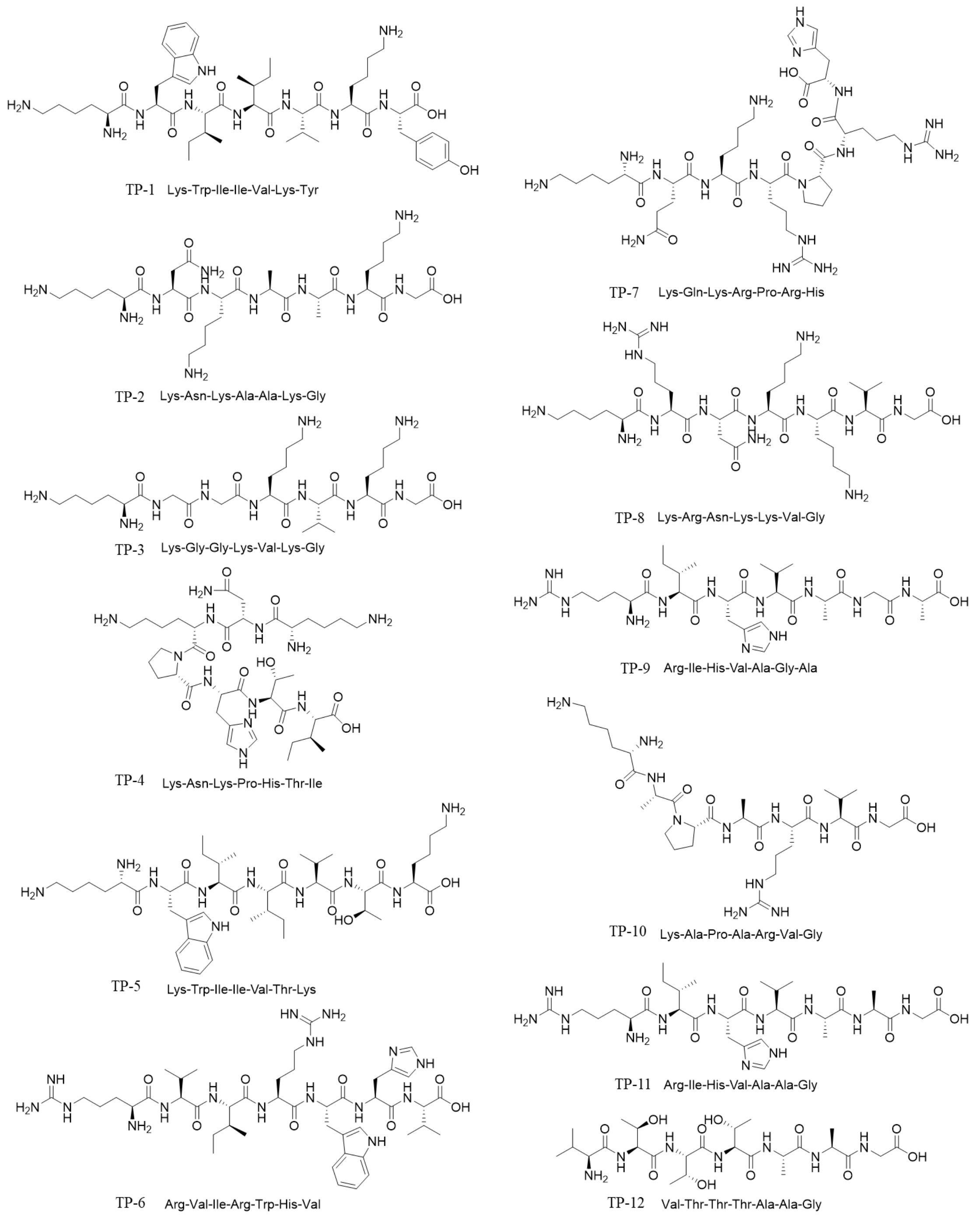

Figure S2. The structures and sequence of TP were unambiguously determined and summarized

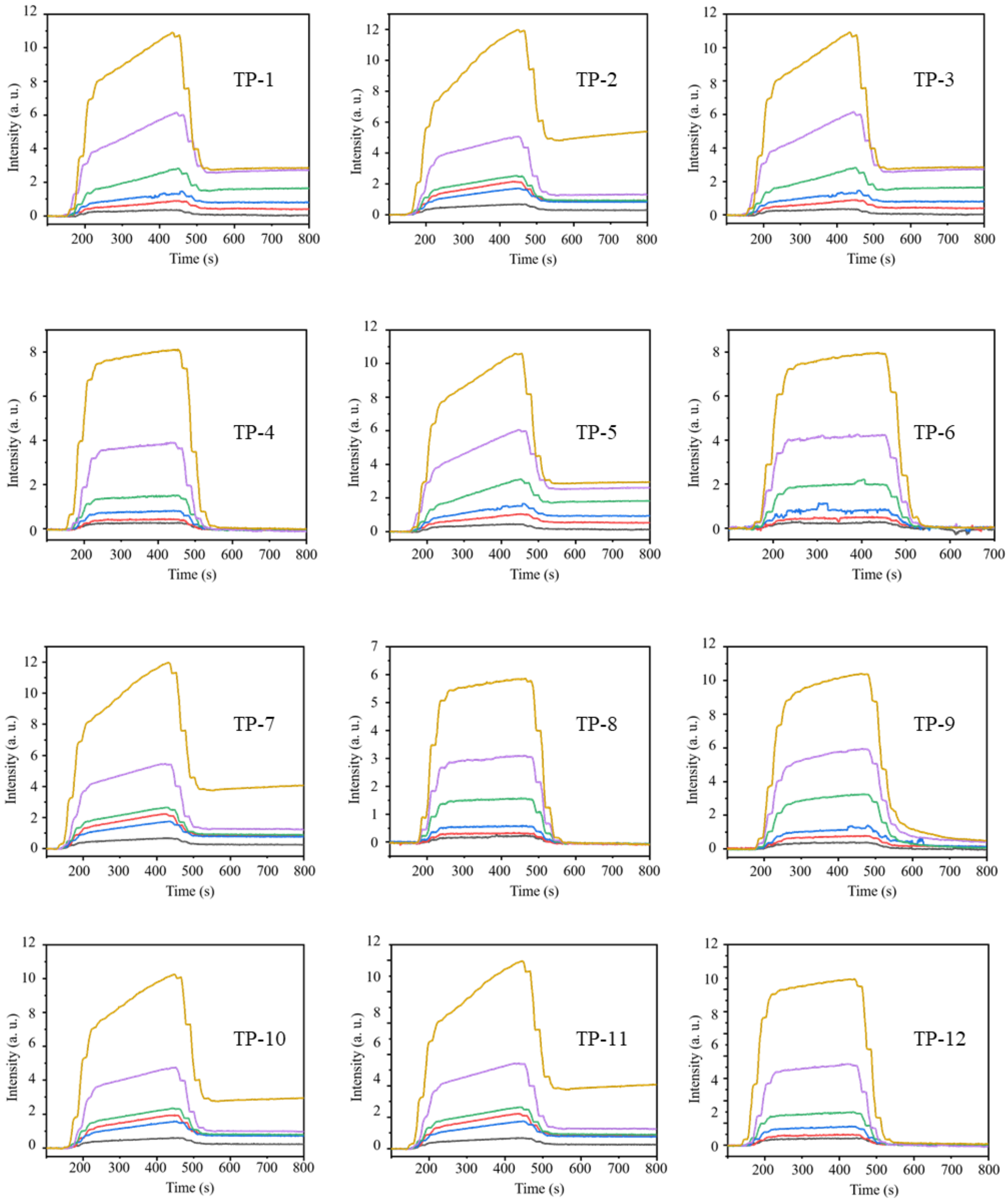

Figure S3. The SPRi curves of TP-1 to TP-12 recognizing Sema4D protein.

MASS SPECTROMETRY REPORT

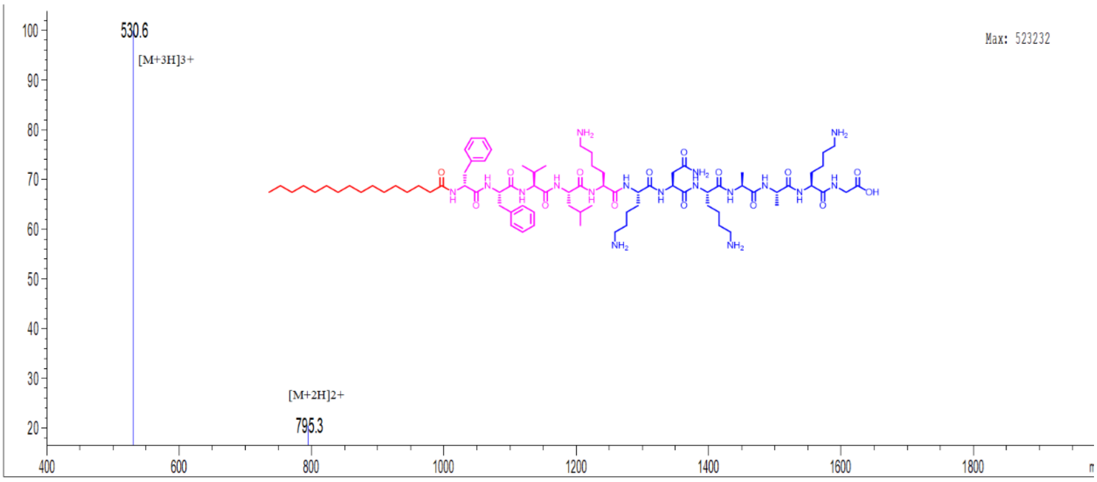

Sample Description		Instrument	Agilent-6125B		
Analyzed date:	2020-08-07	Probe:	ESI	Probe Bias:	+4.5kv
Analyst:	YU	Nebulizer Gas Flow:	1.5L/min	Detector:	1.5kv
Sample:	C16-FFVLK-KNKAAKG	CDL:	-20.0v	T. Flow:	0.2ml/min
M.W.:	1589.10	CDL Temp.:	250 °C	B. Conc.:	50%H2O/50%ACN
Lot. No.:	P200731-XT821942	Block Temp.:	200 °C		

Figure S4. The MADLI-TOF-MS spectrum of SSP-2

REPORT

Instrument No: 0200023
Lot No :P200731-XT821942
Column :4.6*250mm, GS-120-5-C18-BIO
Solvent A :0.1%Trifluoroacetic in 100% Acetonitrile
Solvent B :0.1%Trifluoroacetic in 100% Water

Gradient :	A	B
0.00min	53%	47%
25min	78%	22%
25.1min	100%	0%
30min	Stop	

Flow rate :1.0ml/min
Wavelength :220nm
Volume :10ul

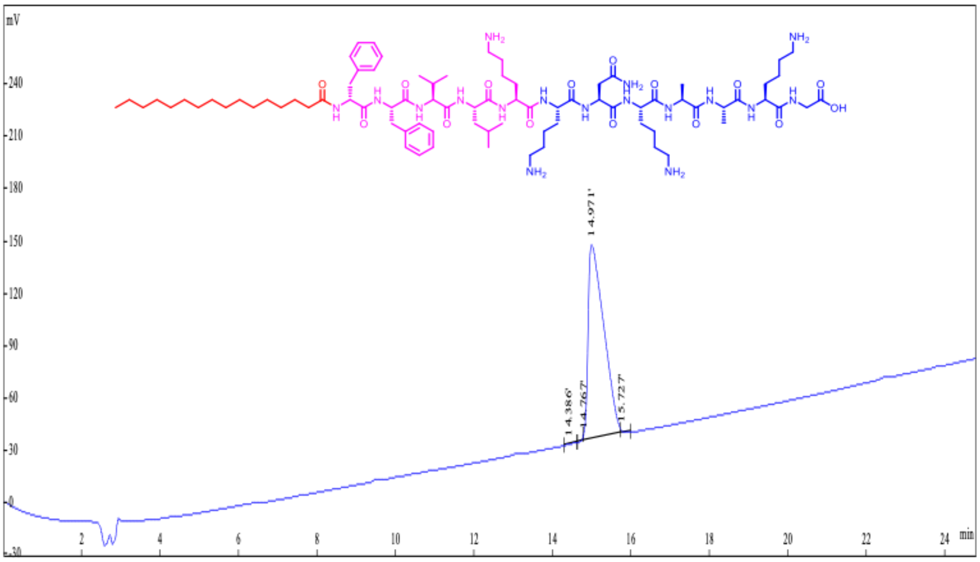

Rank	Time	Conc.	Area	Height
1	14.386	0.0851	2651	244
2	14.767	0.2373	7392	4024
3	14.971	99.3756	3095566	112053
4	15.727	0.3020	9409	2173
Total		100	3115018	118494

Figure S5. The high performance liquid chromatograph spectrum of SSP-2

REPORT

Instrument No: 0200023
Lot No :P200731-XT821943
Column :4.6*250mm,GS-120-5-C18-BIO
Solvent A :0.1%Trifluoroacetic in 100% Acetonitrile
Solvent B :0.1%Trifluoroacetic in 100% Water

Gradient :	A	B
0.00min	50%	50%
25min	75%	25%
25.1min	100%	0%
30min	Stop	

Flow rate :1.0ml/min
Wavelength :220nm
Volume :10ul

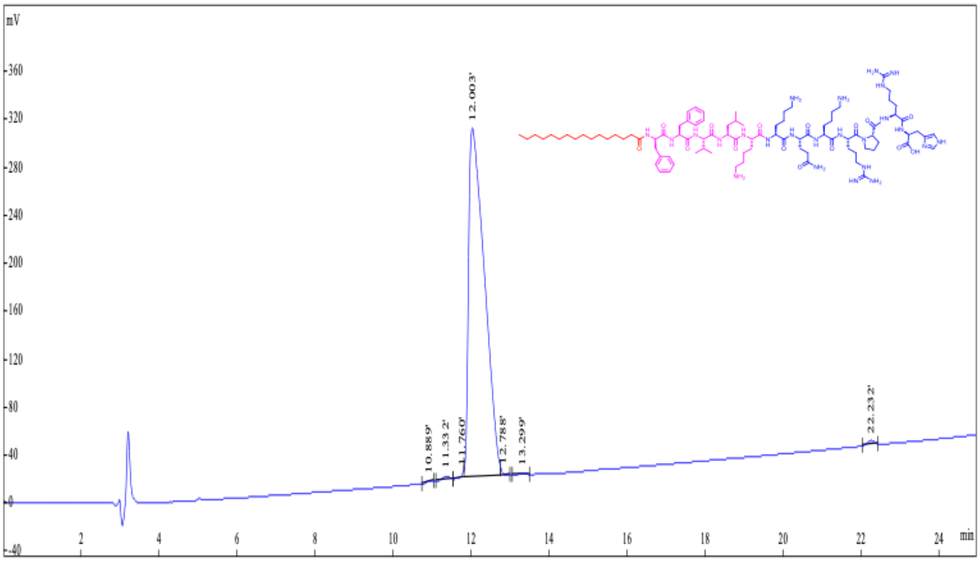

Rank	Time	Conc.	Area	Height
1	10.889	0.1241	10427	1274
2	11.332	0.4368	36693	2754
3	11.760	0.4546	38190	4490
4	12.003	97.9910	8231259	291575
5	12.788	0.2320	19491	2694
6	13.299	0.2367	19883	1532
7	22.232	0.5248	44079	3704
Total		100	8400022	308023

Figure S6. The high performance liquid chromatograph spectrum of SSP-1

MASS SPECTROMETRY REPORT

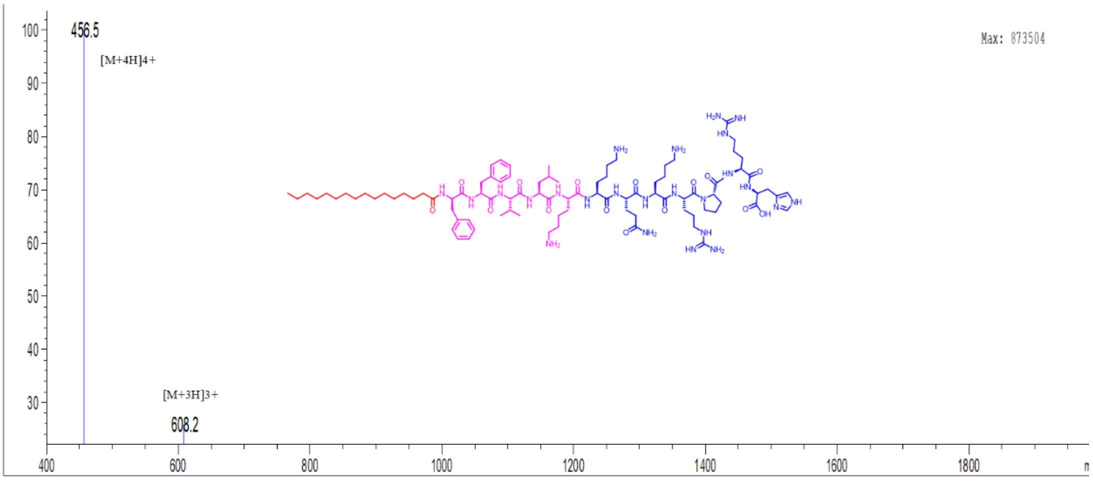

Sample Description		Instrument	Agilent-6125B		
Analyzed date:	2020-08-11	Probe:	ESI	Probe Bias:	＋4.5kv
Analyst:	YU	Nebulizer Gas Flow:	1.5L/min	Detector:	1.5kv
Sample:	C16-FFVLK-KQKRPRH	CDL:	-20.0v	T. Flow:	0.2ml/min
M.W.:	1822.38	CDL Temp.:	250 °C	B. Conc.:	50%H2O/50%ACN
Lot. No.:	P200731-XT821943	Block Temp.:	200 °C		

Figure S7. The MADLI-TOF-MS spectrum of SSP-1

Patients	Sex	Age	Pathology	CST (µm)
**Control 1**	M	59	MH	NA
**Control 2**	F	42	MH	NA
**Control 3**	M	62	MH	NA
**Control 4**	M	55	MH	NA
**Control 5**	M	68	MH	NA
**Control 6**	F	52	MH	NA
**Control 7**	F	48	MH	NA
**Control 8**	M	70	MH	NA
**Control 9**	F	63	MH	NA
**Control 10**	F	55	MH	NA
**Control 11**	F	59	MH	NA
**Control 12**	M	72	MH	NA
**PDR (DME) 1**	F	52	Diabetes (type 2)	506
**PDR (DME) 2**	M	66	Diabetes (type 2)	401
**PDR (DME) 3**	M	58	Diabetes (type 2)	459
**PDR (DME) 4**	F	47	Diabetes (type 2)	439
**PDR (DME) 5**	F	70	Diabetes (type 2)	458
**PDR (DME) 6**	F	63	Diabetes (type 2)	550
**PDR (DME) 7**	M	61	Diabetes (type 2)	486
**PDR (DME) 8**	F	45	Diabetes (type 2)	376
**PDR (DME) 9**	F	54	Diabetes (type 2)	399
**PDR (DME) 10**	M	64	Diabetes (type 2)	338
**PDR (DME) 11**	M	49	Diabetes (type 2)	361
**PDR (DME) 12**	M	66	Diabetes (type 2)	570
**PDR (DME) 13**	F	68	Diabetes (type 2)	574
**PDR (DME) 14**	M	63	Diabetes (type 2)	477
**PDR (DME) 15**	F	70	Diabetes (type 2)	577
**PDR (DME) 16**	F	68	Diabetes (type 2)	664
**PDR (DME) 17**	F	50	Diabetes (type 2)	598
**PDR (DME) 18**	M	74	Diabetes (type 2)	526
**PDR (DME) 19**	M	55	Diabetes (type 2)	489

Table 1. Table of patients used for western blot and Elisa.

This study was approved by an ethics committee of Tongji Medical College, Huazhong University of Science and Technology, Wuhan, China ([2018]伦申字(S477)). PDR: Proliferative Diabetic Retinopathy, DME: Diabetic Macular Edema, MH: Macular Hole, CST: Central Subfield Thickness
